# Analysis of Mn^2+^ and Zn^2+^ Ions in Macroalgae with Heteroelement-Doped Carbon-Based Fluorescent Probe

**DOI:** 10.3390/bios12050359

**Published:** 2022-05-22

**Authors:** Hui Xu, Xin You, Yue Lu, Peng Liang, Zhihui Luo, Yiwei Wang, Shaoxiao Zeng, Hongliang Zeng

**Affiliations:** 1 Engineering Research Centre of Fujian-Taiwan Special Marine Food Processing and Nutrition, Ministry of Education, Fuzhou 350002, China; zsxfst@fafu.edu.cn (S.Z.); zhlfst@fafu.edu.cn (H.Z.); 2 Fujian Provincial Key Laboratory of Quality Science and Processing Technology in Special Starch, Fujian Agriculture and Forestry University, Fuzhou 350002, China; 3 College of Food Science, Fujian Agriculture and Forestry University, Fuzhou 350002, China; 3210910056@fafu.edu.cn (X.Y.); luyueeee@fafu.edu.cn (Y.L.); liangpeng0412@fafu.edu.cn (P.L.); wangyw@fafu.edu.cn (Y.W.); 4 Guangxi Key Laboratory of Agricultural Resources Chemistry and Biotechnology, College of Chemistry and Food Science, Yulin Normal University, Yulin 537000, China; zhluo@ylu.edu.cn

**Keywords:** red carbon dots, manganese ion, zinc ion, fluorescence, macroalgae

## Abstract

Kelp and laver are large economic macroalgae in China, which are rich in nutrients, especially Mn and Zn. Excessive intake of Mn and Zn can be harmful to the human body. Therefore, it is necessary to develop a convenient and efficient method to detect the contents of Mn and Zn in macroalgae. In this experiment, red carbon dots (R-CDs) doped with N and S elements were prepared by the thermal solvent method. The obtained R-CDs displayed excitation wavelength-independent fluorescent emission in the red spectral region. The R-CDs were used to construct a fluorescent probe for specific recognition of Mn^2+^ and Zn^2+^, achieving high-sensitivity detection of Mn^2+^ and Zn^2+^. The detection results showed a good linear relationship between fluorescence intensity and Mn^2+^ concentration, and the calculated detection limit was 0.23 nmol/L. For the detection of Zn^2+^, the detection limit was estimated as 19.1 nmol/L. At the same time, the content distribution of Mn and Zn elements in macroalgae produced in Fujian was investigated by the constructed fluorescence probe. It was found that kelp, laver, and their products are rich in Mn and Zn elements, and the content of Mn and Zn elements in laver is higher than that in kelp, which can be used as the optimal food supplement for Mn and Zn elements.

## 1. Introduction

China is rich in seaweed resources, among which macroalgae are abundant in various nutrients necessary for the human body and have important economic value. Macroalgae have a strong adsorption capacity for heavy metals. The heavy metal content in all kinds of macroalgae is higher than that of water, and the enrichment coefficient of heavy metal elements varies from several times to hundreds of thousands of times [1]. Thus, macroalgae can be used not only as large nutrient reservoirs, but also as research samples for heavy metal pollution investigations. Through previous investigations, it has been found that the contents of Mn and Zn in macroalgae are very high. Mn and Zn, as essential elements, are of great significance to various physiological activities of the human body. However, excessive intake of Mn [2] and Zn [3] will also cause harm to the human body. Therefore, it is necessary and meaningful to establish a convenient analysis method for the detection of Mn^2+^ and Zn^2+^ in macroalgae.

So far, many analytical methods such as atomic absorption spectrometry (AAS), inductively coupled plasma mass spectrometry (ICP-MS), and electrochemistry have been applied to the detection of Mn^2+^ and Zn^2+^. However, these traditional detection methods all have the defects of complex operation and slow response time, and it is urgent to develop a method that is convenient to operate and quickly detect target elements. The fluorescent probe has attracted much attention because of its advantages of good selectivity, convenience, easy pretreatment, live-cell imaging use [4,5], and rapid detection of a variety of metal ions. At present, fluorescence probes commonly used in the detection of metal ions include organic small molecules, metal–organic frameworks (MOFs), aptamers, and nanomaterials. Organic fluorescent molecules, such as rhodamine, coumarin, naphthalimide, benzothiazole, pyridine, and other typical fluorescent groups, have been widely used in the detection of metal ions in food. Yu et al. [6] designed a new coumarin-based ratiometric fluorescent probe using dithiothreitol as the recognition receptor for Hg^2+^ detection based on the change in the color of the fluorescent probe from light yellow to orange. Zhou et al. [7] designed and synthesized a novel fluorescence sensor based on fluoropyrrole and carboxyl mercaptan metal-bonded receptors for the detection of Hg^2+^. The sensor was highly selective for Hg^2+^, the detection limit of Hg^2+^ was 5.7 nM, and the sensor responded quickly to Hg^2+^ in aqueous solution in 30 s. However, poor solubility, serious photobleaching, poor bioavailability, and narrow excitation of these organic fluorescent dyes greatly limit detection and sensing. MOFs are hybrid crystalline porous materials, usually composed of inorganic nodes (metal ions or metal clusters) [8] and functional organic linkers [9,10]. However, due to the instability of many MOF materials in water [11], most current MOF-based fluorescence sensing research on metal ions is carried out in organic solvents, which are not suitable for the practical application of detection probes.

Carbon dots (CDs) have many advantages compared with traditional fluorescent materials, such as unique optical properties, excellent biocompatibility, excellent water dispersibility, low cost, high sensitivity, and easy surface functionalization [12,13]. These advantages make it possible to have great potential application in biomedical imaging and sensing [14], tumor therapy [15], light-emitting devices [16,17], and other fields. Much research has been conducted to synthesize CDs using chemicals [18] or natural products [19] as carbon sources. Xu et al. [20] reported a facile method to prepare copper-doped carbon dots (Cu-CDs) using citric acid and cuprous chloride as precursors under hydrothermal conditions, which showed strong luminescence performance at 440 nm and with 9.81% photoluminescence quantum yield. Cu-CDs with a detection limit of 1 nM can be used for the rapid detection of Fe^3+^, and can be further applied to the detection of Fe^3+^ in human serum. Liu et al. [21] reported that Bombyx mori silk was used as a raw material in the coupling of citric acid to prepare nitrogen-doped CDs using a facile one-step hydrothermal route. The as-prepared nitrogen-doped CDs emitted blue fluorescence with a quantum yield of 61.1%, which can easily bind with Fe^3+^ as a consequence of fluorescence quenching, making a method for Fe^3+^ detection developed with high selectivity and sensitivity. He et al. [22] developed a hydrothermal method to synthesize Zn^2+^-doped carbon quantum dots (Zn-CQDs) using zinc citrate chelate, with a fluorescence quantum yield of 48%. Zn-CQDs have stronger fluorescence sensing ability for Fe^3+^ and Hg^2+^. They can be used in the fields of fluorescence sensors, biological imaging, photoelectronics, and catalysis. However, most of the CDs that have been synthesized have strong emission in the blue-green electromagnetic radiation region and weak emission in the red region with a low quantum yield. Due to the interference of the inherent blue self-fluorescence in the biological matrix, it is necessary to avoid blue emission during practical sample detection. Because of the low extinction coefficient of the biological matrix in the red and near-infrared range in biosensing, red carbon dots (R-CDs) will inevitably show a large signal-to-noise ratio, which is beneficial to improve the sensitivity and accuracy of practical sample detection. Therefore, fluorescent biological probes prepared by using R-CDs can not only effectively avoid self-fluorescence interference, but also have strong penetrability to tissues [23,24], which makes it particularly important and urgent to prepare an R-CDs-based fluorescence probe.

In this paper, R-CDs with dual-emission properties were prepared by the thermal solvent method. The surface morphology and photoluminescence characteristics of R-CDs were studied. The fluorescence probe was constructed by using the R-CDs with dual emission to detect Mn^2+^ and Zn^2+^. Its detection performance was analyzed. Finally, the constructed fluorescent probe was used to detect macroalgae produced in Fujian province, China. The accuracy of the method was verified by flame atomic absorption spectrometry (FAAS). The content of Mn and Zn elements in kelp (*Laminaria Japonica*) and laver (*Porphyra haitanensis*) samples was analyzed, and the proposed fluorescent probe could have potential application in dietary guidance and safety assessment.

## 2. Materials and Methods

### 2.1. Preparation of R-CDs

The R-CDs were prepared by the thermal solvent method, which can be described as follows: 0.6804 g reduced glutathione (Macklin, Shanghai, China) and 0.6106 g o-phenylenediamine (Merck, Darmstadt, Germany) were dissolved in 20 mL of formamide (Macklin, Shanghai, China) at room temperature. The obtained mixture was transferred into a Teflon-lined stainless steel autoclave, which was then kept in an air-circulating oven at 160 °C for 2 h. After the reaction, the autoclave was naturally cooled down to room temperature. The obtained solution was centrifuged at the speed of 1000 rpm for 20 min to remove large particles. The upper solution was diluted and dialyzed (molecular weight cut-off = 1000 Da) against ultrapure water for a week. The purified R-CDs solution was freeze-dried to obtain dark green powder for characterization. Ultrapure water (DI, >18.25 MΩ) was prepared by the Millipore Milli-Q Water Purification System (Merck, Billerica, MA, USA) for the preparation of all solutions in this work.

### 2.2. Structural Characterization of R-CDs

Fourier transform infrared spectroscopy (FT-IR) was obtained using a VERTEX 80V FT-IR spectrometer (Bruker, Billerica, MA, USA). FT-IR samples were prepared by mixing the powders of KBr and R-CDs in a ratio of 1:150. The obtained mixture was made into tablets. TEM (Shimadzu, Tokyo, Japan) samples were prepared by dropping the aqueous solution containing R-CDs onto carbon-coated grids and allowing the excess solvent to evaporate. The structure of the as-prepared R-CDs was characterized by XRD-6000 (Shimadzu, Tokyo, Japan). The fluorescence measurements were performed with a fluorescence spectrophotometer RF-5301PC (Shimadzu, Tokyo, Japan). The samples were excited from 360 to 420 nm, and the emission spectrum in the range of 220 to 900 nm was measured. The slit width was fixed at 10 nm for emission. The ultraviolet–visible (UV–VIS) spectra were recorded on a UV-2600 ultraviolet–visible spectrophotometer (Shimadzu, Tokyo, Japan), with scanning wavelength ranging from 200 nm to 800 nm using a xenon lamp and a tungsten lamp.

### 2.3. Signal-Off Detection of Mn^2+^

For the detection of Mn^2+^, 1 mL of purified R-CDs solution and varying concentrations of Mn^2+^ ion standard solution (Macklin, Shanghai, China) were separately added into the mixture of 980 μL of 0.1 M HEPES buffer solution (Solarbio, Beijing, China). Then, the mixture was thoroughly shaken and equilibrated at room temperature. The fluorescence spectra with 420 nm excitation wavelength and 680 nm emission wavelength were recorded and used for quantitative analysis.

### 2.4. Ratiometric Detection of Zn^2+^

For the detection of Zn^2+^, 1 mL of purified R-CDs solution and varying concentrations of Zn^2+^ ion standard solution (Macklin, Shanghai, China) were separately added into the mixture of 980 μL of 0.1 M HEPES buffer solution (Solarbio, Beijing, China). Then, the mixture was thoroughly shaken and equilibrated at room temperature. The fluorescence spectra with excitation at 420 nm and emission at 650 nm and 680 nm were recorded and used for quantitative analysis.

### 2.5. Sample Pretreatment

In this work, 20 different brands of macroalgae samples were purchased from supermarkets and retailers in Fujian province. Four hundred milligrams of the cleaned and dried samples was ground into powder, added with 5 mL HNO_3_ into the microwave digestion tank, and digested with an 800 W microwave digestion instrument. The digested sample was heated up to 140–160 °C to remove excess acid. After cooling to room temperature, approximately 1 mL of sample solution remained and was transferred to a volumetric flask with ultrapure water to 25 mL. The sample solution could be diluted with need. Blank samples were prepared according to the same method without adding macroalgae.

### 2.6. FAAS Determination

A series of standard concentrations of Mn solution and Zn solution were prepared. The absorbance is plotted as the function of metal element concentration to obtain a calibration curve for quantitative analysis. The pretreated sample was placed in FAAS (AA-6300C, Shimadzu, Tokyo, Japan), and the contents of Mn and Zn elements were detected according to the test parameters of different elements. Among them, the test parameters for Mn detection were lamp current 2.0 mA, wavelength 279.5 nm, slit 0.1 nm, negative high pressure −344 V, air flow 6.0 L·min^−1^, acetylene flow 1.0 L·min^−1^. The test parameters for Zn detection were lamp current 2.0 mA, wavelength 213.9 nm, slit 0.2 nm, negative high pressure −309 V, air flow 6.0 L·min^−1^, and acetylene flow 1.0 L·min^−1^.

## 3. Results and Discussion

### 3.1. Photoluminescence Performance of R-CDs

**Figure 1:**
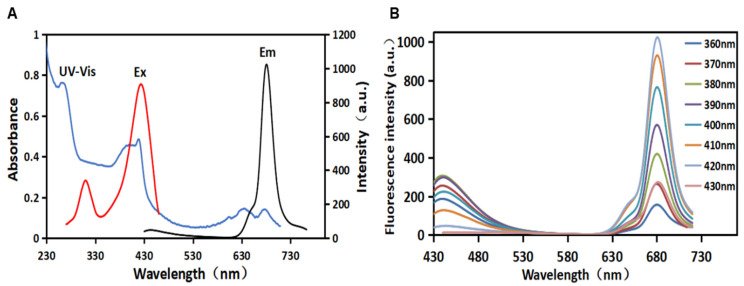
UV–visible absorption spectrum (blue), excitation spectrum (red), and emission spectrum (black) of R-CDs (**A**); fluorescence emission spectra of R-CDs (**B**).

As shown in Figure 1A, the UV–VIS absorption peak of the synthesized CDs absorption spectrum at 270 nm could be attributed to the π–π* transition of the conjugated aromatic sp^2^ bonds [25,26]. The absorption spectrum of CDs also displayed several characteristic absorbing peaks at 395 nm, 420 nm, 640 nm, and 680 nm, which could be attributed to the n–π* and π–π* transitions of the aromatic π system containing the C=O, C=N, and C=S bonds, respectively [27]. These heteratomic groups could form polycyclic aromatic hydrocarbon or oxygen-containing structures on the surface of CDs. Additionally, the CDs had an excitation peak at 420 nm and an emission peak at 680 nm. The fluorescence excitation band of CDs overlapped with its main UV–VIS absorption band, indicating that the emission was closely related to these absorption bands caused by related structures [28]. The maximum absorption wavelength of CDs was used as the fluorescence excitation wavelength to obtain the fluorescence emission spectrum of CDs. Figure 1B shows the emission spectrum of the dual-emission fluorescence CDs. The quantum yield of R-CDs was calculated to be 13.64%. Under single-wavelength excitation, there were two maximum emission peaks in the blue (λ_Em_ = 442 nm) and red (λ_Em_ = 680 nm) regions. When the excitation wavelength increases from 360 nm to 430 nm, the two fluorescence emission peaks do not shift. It was indicated that the synthesized CDs showed excitation wavelength-independent fluorescent emission. This characteristic may be related to the surface state or the uniform particle size of CDs, which can avoid the interference of autoluminescence in application. As λ_Ex_ increases from 360 nm to 430 nm, the fluorescence intensity at λ_Em_ 442 nm and 680 nm increases and then decreases. When λ_Ex_ = 380 nm, the fluorescence intensity at λ_Em_ = 442 nm is the strongest. When λ_Ex_ = 420 nm, the fluorescence intensity at λ_Em_ = 680 nm is the strongest. Under short-wavelength light excitation, CDs have two characteristic fluorescence emission peaks in the blue and red regions of the spectrum. These dual-fluorescence bands observed may be attributed to core- and surface-state emission [29]. Being capable of excitation at different wavelengths makes the CDs attractive for a variety of applications. In this case, red fluorescence (λ_Em_ = 680 nm) was selected in biological studies to reduce the interference caused by biomolecules’ self-fluorescence, resulting in minimal light damage and deeper tissue penetration [30]. Therefore, the red carbon dots (R-CDs) prepared in this work were used as fluorescent probes for metal element detection.

### 3.2. Structural Characterization of R-CDs

**Figure 2:**
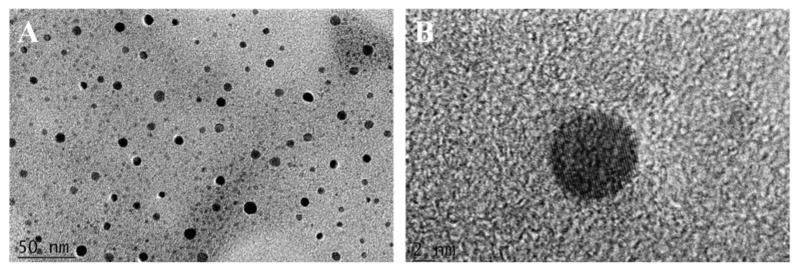
TEM (**A**) and HRTEM images (**B**) of R-CDs.

The morphology and size of R-CDs were observed by TEM. As can be seen from Figure 2A, R-CDs showed good dispersion, no agglomeration, and the morphology was quasi-spherical. The average particle size of R-CDs was estimated as 5.46 ± 1.03 nm by counting the size of more than 100 particles, without any large particles or aggregation. It was indicated that the reaction process was stable, and no adverse reactions occurred. Figure 2B shows clear lattice fringes with a spacing of 0.32 nm, corresponding to the 002 crystal plane of graphite carbon, indicating that the material contained a graphite-like structure [31].

**Figure 3:**
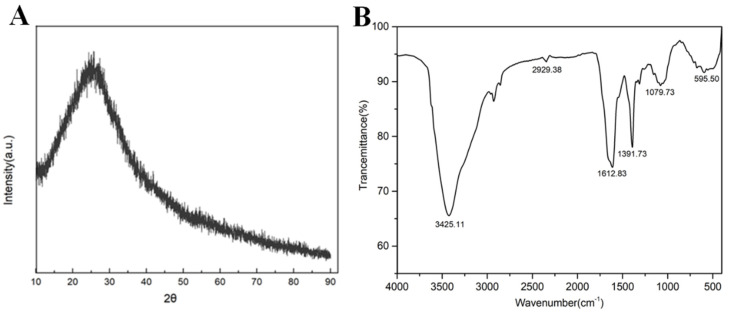
XRD (**A**) and FT-IR spectra of R-CDs (**B**).

R-CDs were characterized by XRD. As shown in Figure 3A, R-CDs had a wide diffraction peak at 2θ = 26° corresponding to plane 002 of the sp^2^ hybrid ink carbon, which was consistent with the TEM results [31]. The FT-IR spectrum of R-CDs is shown in Figure 3B. A wide FT-IR absorption band appeared at 3000~3500 cm^−1^, which was caused by the stretching vibration of the O-H bond and the N-H bond [24]. Two characteristic absorption peaks appeared at 1079 cm^−1^ and 1391 cm^−1^, which were caused by the stretching vibration of the C-O bond and the C-N bond. The peak at 1612 cm^−1^ was caused by the C=O stretching vibration [32]. The results show that there were abundant functional groups on the surface of R-CDs, such as amino, hydroxyl, and carboxyl groups, demonstrating the good hydrophilicity of R-CDs.

The XPS spectra were obtained using a GENESIS 60S XPS (EDAX, Warrendale, PA, USA). Figure S1 shows the full spectrum of XPS. There were four characteristic peaks of R-CDs, corresponding to the C1s peak at 284.6 eV, the O1s peak at 530.25 eV, the N1s peak at 398.97 eV, and the S2p peak at 162.1 eV, and the contents of corresponding elements were 56.56%, 24.67%, 16.12%, and 2.65%, respectively. The results show that N and S can be doped into R-CDs by changing the composition and proportion of synthetic materials. Photoelectron spectrum C1s (Figure S2A) shows that C1s was fitted by three peaks located at 284.82 eV, 288.27 eV, and 286.12 eV, corresponding to C-C/C=C, -COOH, and C-O/C-N respectively. Photoelectron spectrum O1s (Figure S2B) showed that O1s was fitted by two peaks, corresponding to 531.37 eV C=O and 532.87 eV C-OH, respectively. N1s (Figure S3C) was fitted to obtain four main peaks of 398.52 eV, 399.57 eV, 400.12 eV, and 401.07 eV, corresponding to C=N, pyridine N, pyrrole N, and graphite N, respectively. The peaks of S2p (Figure S3D) at 161.12 eV and 163.22 eV were attributed to S2p1/2, S2p1/2, and S2p3/2. Peaks at 163.67 eV and 164.82 eV were classified as -C-SO and -C-SO_2_. R-CDs had a conjugated Sp2 domain rich in oxygen/nitrogen surface groups. Nitrogen could use the unpaired electrons to improve the emission characteristics of R-CDs. The energy gap regulation in R-CDs also depended on the N and O content. The oxygen-containing functional groups, particle size, and graphite nitrogen determined the fluorescence characteristics of R-CDs. Zeta potential showed that the surface potential of R-CDs was −20 eV, demonstrating a strong negative charge and containing a large number of oxygen carboxyl groups on the surface. The more carboxyl groups on the surface of R-CDs or the higher oxidation degree, the R-CDs more easily emitted in the red spectral region.

### 3.3. Stability of R-CDs

The pH, ionic strength, and UV lamp irradiation were investigated for the study of R-CDs stability. The pH value affected the emission of R-CDs. As shown in Figure S3, the fluorescence intensity of R-CDs in acidic solution increased upon enhancing the pH value. Under the alkaline condition, the red emission spectra can be deconvoluted into two peaks at 650 nm and 680 nm. As the pH value increased, peak intensity dominated at 650 nm, and the opposite effect was observed at lower pH. This phenomenon was estimated to be caused by the chemical properties of surface functional groups of R-CDs that affected their electronic properties. The pH-dependent fluorescence was associated with protonation of amino groups, deprotonation of carboxyl groups, or tautomerism of amides. As shown in Figure S4A, when the concentration of NaCl reached 1 mol/L, the fluorescence intensity was not significantly affected, indicating that R-CDs had good stability in the high-ionic-intensity environment. The fluorescence intensity of R-CDs was measured by a fluorescence spectrophotometer after being irradiated under an excitation UV lamp at 365 nm for 90 min (Figure S4B), and it was found that the fluorescence intensity decreased by only about 15%. This indicated that R-CDs have good photobleaching resistance.

### 3.4. Photoluminescence Mechanism of R-CDs

**Figure 4:**
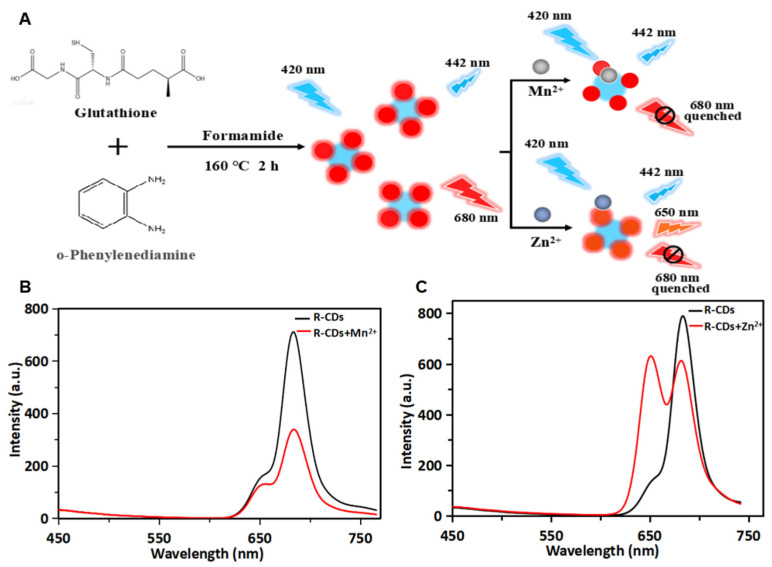
Schematic illustration of the preparation of R-CDs and the application in the detection of Mn^2+^ and Zn^2+^ (**A**). Fluorescence spectra of R-CDs (black curve) and R-CDs+Mn^2+^ (red curve) (**B**). Fluorescence spectra of R-CDs (black curve) and R-CDs+Zn^2+^ (red curve) (**C**).

As shown in Figure 4A, R-CDs, as fluorescent probes, were synthesized using glutathione and o-phenylenediamine as raw materials and formamide as solvent to detect metal elements. The incorporation of metal elements into carbon-based nanomaterials could enhance the conductivity and electrical capacitance. The metal-decorated nanomaterials exhibited a saturated absorption process of optical nonlinearity by near-resonant energy transfer [33]. Therefore, the proposed carbon-based fluorescent probe was applied to investigate the interaction between carbon dots and Mn^2+^/Zn^2+^, and the possible photoluminescence mechanism was discussed. The fluorescence spectra of R-CDs and R-CDs mixed with Mn^2+^ are shown in Figure 4B. It was found that the presence of Mn^2+^ could effectively quench the fluorescence of R-CDs at 680 nm. The average fluorescence lifetimes (τ) were calculated to be 4.38 ns for R-CDs and 4.37 ns for R-CDs + Mn^2+^. This indicated that the fluorescence quenching was caused by the static quenching. This phenomenon may be related to the coordination of -NH_2_ on the surface of R-CDs and Mn^2+^. Electrons transferred from the excited state of R-CDs to the unfilled orbital of Mn^2+^, forming a new electron–hole recombination, which was consistent with a previous report [34]. It can promote the nonradiative recombination process of excitons through an effective electron transfer process, and finally quench the fluorescence of R-CDs at 680 nm. The spectra result of Zn^2+^ mixed with R-CDs showed that the presence of Zn^2+^ led to a newly generated fluorescence peak at 650 nm and quenching of the fluorescence peak at 680 nm (Figure 4C). The average fluorescence lifetimes (τ) were calculated to be 3.20 ns for R-CDs and 3.25 ns for R-CDs + Zn^2+^. The slightly changed lifetime indicated that the fluorescence quenching was caused by the static quenching. The quenching was attributed to the chelation between Zn^2+^ with nitrogen and oxygen atoms and nonradiative recombination through the charge transfer [35]. The 30 nm blue shift of R-CDs after adding Zn^2+^ might be due to the coordination-induced surface passivation [36,37].

### 3.5. Signal-Off Analysis of Mn^2+^

**Figure 5:**
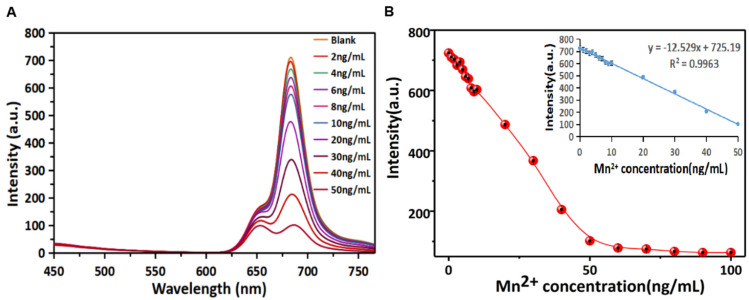
Fluorescence responses of the R-CDs upon the addition of different concentrations of Mn^2+^ (**A**) and the linear correlation of fluorescence intensity versus the concentrations of Mn^2+^ in the range from 0 ng/mL to 100 ng/mL (**B**).

Based on the quenching of R-CDs fluorescence by Mn^2+^, a method for the rapid detection of Mn^2+^ in the “signal-off” mode was established. Under the optimal detection conditions (see Supplementary Materials and Figure S5 for details), the fluorescence quenching ability of R-CDs with different concentrations of Mn^2+^ was investigated. Figure 5A records the fluorescence spectra of R-CDs when Mn^2+^ with different concentrations (2–50 ng/mL) was added. It can be seen that the fluorescence intensity at 680 nm was the maximum when no target element existed. The emission of R-CDs gradually decreased with the increase in Mn^2+^ concentration, showing a good quenching effect. As shown in Figure 5B, the fluorescence intensity could be completely quenched when the concentration of Mn^2+^ exceeded 90 ng/mL. It can be clearly seen that the fluorescence intensity was inversely proportional to the Mn^2+^ concentration in the range of 1~50 ng/mL, and the linear equation was y = −12.296x + 725.44 with R^2^ = 0.999. The detection limit of the method was estimated to be 0.0127 ng/mL (equivalent to 0.23 nmol/L). In this work, R-CDs were synthesized by the thermal solvent method at one time. The operation was simple, and the R-CDs surface was rich in functional groups containing nitrogen and oxygen, so Mn^2+^ can be specifically detected without additional chemical modification. Compared with other published methods for Mn^2+^ detection (Table 1), R-CDs-based signal-off analysis of Mn^2+^ showed a lower detection limit and higher sensitivity.

**Figure 6:**
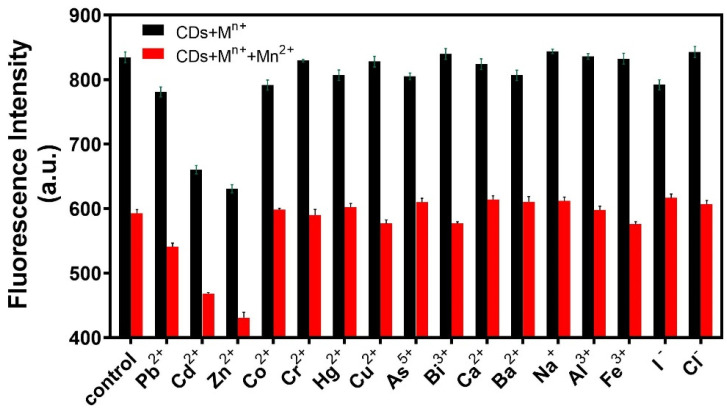
Specificity of the fluorescence probe for Mn^2+^ analysis.

Specificity is also an important factor in the evaluation of the analytical performance of fluorescent probes. Sixteen interference ions were selected for specificity experiments. A certain amount of R-CDs solution was mixed with 20 μL of interfering ions (20 ng/mL), and the changes in fluorescence intensities were recorded under the same conditions. As shown in Figure 6, when R-CDs were mixed with other interfering ions, the fluorescence intensity of the (R-CDs + M^n+^) did not change significantly compared with the fluorescence intensity of R-CDs. However, when Mn^2+^ was added to the (R-CDs + M^n+^) mixture, the fluorescence intensity decreased significantly, which was consistent with the change in fluorescence intensity of (R-CDs + Mn^2+^). The results show that the fluorescent probe had good selectivity for Mn^2+^. Among all kinds of interference ions, Cd^2+^ and Zn^2+^ showed different degrees of interference. In the real macroalgae sample, the content of Mn^2+^ and Zn^2+^ is much higher than that of Cd^2+^ [42,43], and the interference caused by Cd^2+^ can be ignored.

### 3.6. Ratiometric Analysis of Zn^2+^

**Figure 7:**
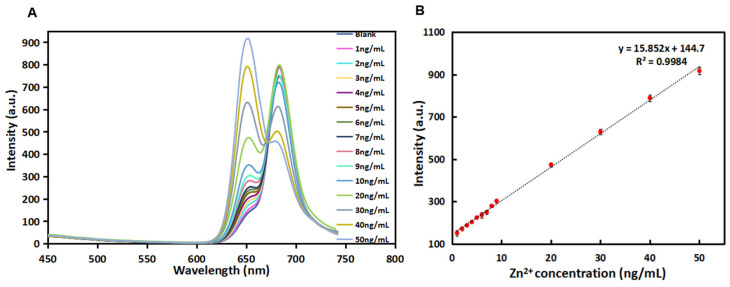
Fluorescence responses in the λ_Em_ = 650 nm of the R-CDs upon the addition of different concentrations of Zn^2+^ (**A**) and linear correlation of fluorescence intensity versus the concentrations of Zn^2+^ in the range from 1 ng/mL to 50 ng/mL (**B**).

Under the optimal detection conditions (see Supplementary Materials and Figure S6 for details), the fluorescence performance of R-CDs with different concentrations of Zn^2+^ was investigated. In Figure 7A, the fluorescence spectra of R-CDs with different concentrations (1–50 ng/mL) of Zn^2+^) are recorded. The intensity at a wavelength of 680 nm displayed negligible change at the low concentration range. When the concentration increased higher than 20 ng/mL, the emission intensity decreased. Meanwhile, it was observed that the fluorescence intensity at 650 nm increased gradually when the concentration of Zn^2+^ increased from 1 ng/mL to 50 ng/mL. As shown in Figure 7B, the fluorescence intensity change at λ_Em_ = 650 nm was proportional to the Zn^2+^ concentration in the range of 1~50 ng/mL, and the linear equation was y = 15.852x + 144.7 with R^2^ = 0.9984. The detection limit of the method was estimated to be 1.25 ng/mL (equivalent to 19.1 nmol/L). Compared with other published methods for Zn^2+^ detection (Table 2), R-CDs analysis of Zn^2+^ shows a lower detection limit and higher sensitivity.

**Figure 8:**
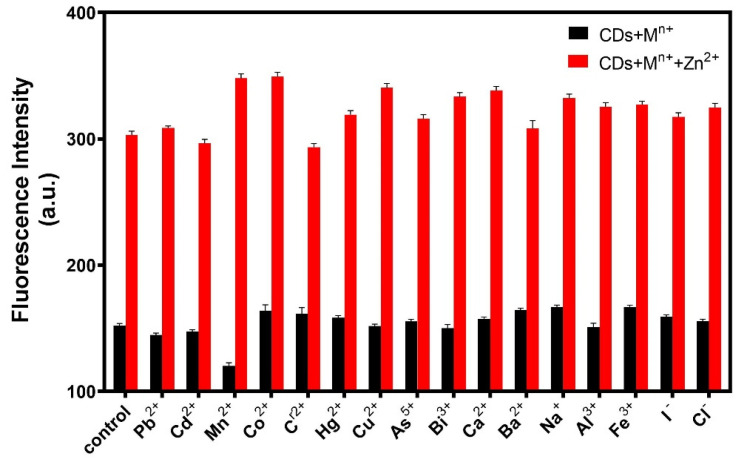
Specificity of the fluorescence probe for sensing Zn^2+^.

To evaluate the specificity of the fluorescent probe, sixteen interference ions were selected. A certain amount of R-CDs solution was mixed with 20 μL of interfering ions (20 ng/mL), and the changes in fluorescence intensities were recorded under the same conditions. As shown in Figure 8, when R-CDs were mixed with other interfering ions, the fluorescence intensity of the (R-CDs + M^n+^) did not change significantly compared with the fluorescence intensity of R-CDs. However, when Zn^2+^ was added to the (R-CDs + M^n+^) mixture, the fluorescence intensity increased significantly, which was consistent with the change of fluorescence intensity of (R-CDs + Zn^2+^). The results show that the fluorescent probe had good selectivity for Zn^2+^.

### 3.7. Detection of Metal Elements in Macroalgae by R-CDs

The content of Mn^2+^ and Zn^2+^ in twenty algal samples was investigated. Sample Nos. 1–8 were laver, Nos. 9–15 were kelp, and Nos. 16–20 were laver products. The R-CDs were used as a fluorescent probe to detect the content of Mn and Zn elements in the samples. FAAS was applied for verification of the proposed method. The results are shown in Table 3 and Table 4. It was found that the detection results of the R-CDs method were consistent with that of the national standard method, demonstrating the good accuracy of the proposed method. The R-CDs could be used as a new detection fluorescence probe to detect Mn and Zn elements.

Based on the results in Table 3 and Table 4, it could be concluded that the average content of Mn^2+^ in laver produced in Fujian was 41.56 mg/kg, and the No. 5 sample produced in Quanzhou showed the highest content of Mn element of 105.96 mg/kg. The average content of Mn^2+^ in kelp was 34.01 mg/kg, and the concentration ranged from 27 to 49 mg/kg. The average Mn^2+^ content of laver products from Fujian was 37.71 mg/kg. After the deep processing, the formation process of the product had no great influence on the content of Mn^2+^.

The average Zn^2+^ content of laver from Fujian was 57.45 mg/kg, and the concentration ranged from 30 to 94 mg/kg. The average content of Zn^2+^ in kelp was 32.27 mg/kg, and the concentration ranged from 18 to 50 mg/kg. The average Zn^2+^ content in laver products was 30.58 mg/kg. The Zn^2+^ content of Nos. 1–4 laver raw materials purchased by supermarkets was higher than that of Nos. 5–8 samples purchased by retailers. The Zn^2+^ content in laver was decreased after processing. The content of Mn^2+^ and Zn^2+^ in laver was higher than that of kelp. The daily Mn^2+^ supply for adults is 2.5 to 5 mg, and the daily Zn^2+^ supply for adults is 15 to 20 mg. Therefore, laver and kelp can be selected as the daily food supplementing of Mn and Zn.

### 3.8. Recovery

The No. 18 sample was selected for standard recovery of Mn^2+^ detection. A certain amount of Mn^2+^ standard solution was added, and the concentrations of samples were adjusted to 0.4 mg/mL, 2 mg/mL, and 4 mg/mL Mn^2+^, respectively. The responses were analyzed by the R-CDs fluorescence probe. The No. 10 sample was selected for standard recovery of Zn^2+^ detection. The results are shown in Table 5 and Table 6. The spiked recoveries of Mn^2+^ were all in the range of 82–120%, with relative standard deviations less than 10%. The recoveries of Zn^2+^ ranged from 97% to 120% with standard deviations less than 10%. The experimental results showed that the R-CDs fluorescence probe had good stability and accuracy for the detection of Mn^2+^ and Zn^2+^.

## 4. Conclusions

In this study, R-CDs were prepared by the thermal solvent method. The structure and properties of the R-CDs were characterized and analyzed. The results showed that the synthesized R-CDs had excitation wavelength-independent fluorescence emission. Mn^2+^ and Zn^2+^ were detected by the R-CDs-based fluorescence probes. It was found that there was a good linear relationship between fluorescence intensity and Mn^2+^ concentration, with a linear range of 1–50 ng/mL and a measuring limit of 0.23 nmol/L. Meanwhile, the fluorescence intensity showed a good linear relationship with the concentration of Zn^2+^. The linear range was 1–50 ng/mL, and the detection limit was 19.1 nmol/L. This method was simple to operate and quick to respond without chemical modification of the probe. The quantitative determination of Mn^2+^ and Zn^2+^ in macroalgae from Fujian province was carried out based on the established fluorescence probe method. The experimental results showed that macroalgae were rich in Mn and Zn elements. It was found that Mn and Zn contents in laver were higher than that in kelp. Through this work, the distribution characteristics of the main metal elements in kelp and laver products in the future were preliminarily understood. It provided basic data for dietary guidance and health evaluation of kelp and laver to support the value of the macroalgae industry.

## Figures and Tables

**Table 1:** Comparison between this method and other methods for the detection of Mn^2+^.

Detection Method	Material	LOD	Linear Range	Ref.
Electrochemical	Mn(II)-IIP/MWCNT-Chit-IL/GC	0.15 μmol/L	2~9 μmol/L	[38]
Colorimetric	Silver nanoparticles	20 nmol/L	0~700 nmol/L	[39]
Fluorescence	Polymer dot	0.4 μmol/L	1.5~100 μmol/L	[40]
Fluorescence	Silicon nanoparticles	0.2 μmol/L	2.5~250 μmol/L	[41]
Fluorescence	R-CDs	0.23 nmol/L	18.2~910 nmol/L	This method

**Table 2:** Comparison between this method and other methods for the detection of Zn^2+^.

Detection Method	Material	LOD	Linear Range	Ref.
Electrochemical	Bismuth—nitride nanocomposites	0.5 μg/L	1~20 μg/L	[44]
Fluorescence	Lanthanide complexes	1.2 μmol/L	-	[45]
Fluorescence	Thiourea based chemical sensor	0.67 μmol/L	-	[46]
Fluorescence	Novel fluorescent peptide-based probe	26.77 nmol/L	-	[47]
Fluorescence	CDs	19.1 nmol/L	1~50 ng/mL	This method

**Table 3:** Comparison of Mn^2+^ content in samples detected by two methods.

No.	R-CDs	RSD	FAAS	RSD
1	34.95	5.13%	34.17	1.19%
2	33.59	6.81%	37.32	0.8%
3	30.11	4.30%	29.30	2.36%
4	35.45	2.69%	26.24	1.62%
5	105.96	4.28%	116.46	2.20%
6	30.96	2.98%	33.00	1.12%
7	32.53	3.54%	49.18	3.99%
8	28.90	6.98%	31.00	0.75%
9	41.55	6.90%	45.60	1.13%
10	32.83	7.24%	28.14	3.18%
11	40.84	5.85%	31.05	1.13%
12	19.88	1.88%	23.44	1.40%
13	27.08	3.27%	31.76	2.58%
14	27.64	5.32%	43.35	2.85%
15	48.26	1.06%	46.90	3.57%
16	26.23	6.55%	24.35	3.47%
17	12.11	4.51%	17.68	0.93%
18	10.52	1.14%	10.44	1.95%
19	20.63	7.79%	25.70	4.07%
20	24.36	4.01%	26.29	1.32%

Concentration unit is mg/kg.

**Table 4:** Comparison of Zn^2+^ content in samples detected by two methods.

No.	R-CDs	RSD	FAAS	RSD
1	85.14	3.02%	105.16	6.03%
2	93.76	5.57%	103.95	1.19%
3	69.89	2.64%	65.46	5.55%
4	79.17	10.06%	88.40	1.23%
5	51.71	3.23%	61.10	1.83%
6	47.18	4.39%	70.07	7.16%
7	52.58	4.75%	61.95	3.98%
8	32.72	2.14%	29.74	0.97%
9	49.00	1.51%	48.07	1.20%
10	18.30	0.91%	15.82	4.15%
11	25.83	1.13%	28.92	1.69%
12	33.81	5.79%	31.98	3.68%
13	34.21	1.16%	47.01	1.01%
14	33.79	2.62%	44.01	1.30%
15	30.92	4.01%	28.30	2.76%
16	18.75	3.97%	28.04	6.19%
17	26.22	0.79%	32.27	2.40%
18	27.69	0.23%	19.57	3.25%
19	36.11	4.21%	36.45	2.65%
20	44.14	1.36%	49.03	5.34%

Concentration unit is mg/kg.

**Table 5:** Recovery of fluorescent probe detecting Mn^2+^.

Sample	Original Content	Add Scalar	Final Content	Recovery	Recovery Rate	RSD
blank	0.002	0.4	0.4002	0.409~0.432	102.2~107.9%	7.6%
blank	0.002	2	2.002	2.136~2.36	106.7~117.7%	3.5%
blank	0.002	4	4.002	3.30~3.483	82.4~95.7%	9.47%
18	0.168	0.4	0.568	0.558~0.594	98.3~104.6%	2.19%
18	0.168	2	2.168	1.807~2.193	83.3~101.2%	7.0%
18	0.168	4	4.168	4.205~4.393	100.9~105.4%	1.73%

Concentration unit is mg/mL.

**Table 6:** Recovery of fluorescent probe detecting Zn^2+^.

Sample	Original Content	Add Scalar	Final Content	Recovery	Recovery Rate	RSD
blank	0.0577	0.4	0.4577	0.461~0.475	100.7~103.7%	1.16%
blank	0.0577	2	2.0577	2.115~2.141	102.9~104.0%	2.01%
blank	0.0577	4	4.0577	3.943~4.120	97.2~101.53%	8.81%
10	0.293	0.4	0.693	0.734~0.751	106.1~108.4%	2.59%
10	0.293	2	2.293	2.719~2.722	118.5~118.7%	5.28%
10	0.293	4	4.293	4.158~4.201	98.09~99.1%	3.87%

Concentration unit is mg/mL.

## Data Availability

Not applicable.

## Supplementary Materials

The following supporting information can be downloaded at: www.mdpi.com/article/10.3390/bios12050359/s1, Figure S1: Full XPS spectra of R-CDs. Figure S2: XPS C1s (A), O1s (B), N1s (C), and S2p (D) spectra of R-CDs. Figure S3: Effect of pH on the fluorescence emission of R-CDs. Figure S4: Fluorescence intensity changes of R-CDs in different concentration solutions of NaCl (A) and under continuous UV illumination (B). Figure S5: Optimization of pH value (A), buffer solution type (B), and reaction time (C). Figure S6: Optimization of buffer solution type (A) and reaction time (B).
