# Supplementary material for: Analysis of Mn2+ and Zn2+ Ions in Macroalgae with Heteroelement-Doped Carbon-Based Fluorescent Probe
Source: Biosensors (Basel). 2022 May 22;12(5):359. doi: 10.3390/bios12050359 (PMC9138788; doi:10.3390/bios12050359)
Supplement: Supplementary file 1 [file biosensors-12-00359-s001.zip › biosensors-1718578-supplementary.pdf]

biosensors

Supplementary Materials

Analysis of Mn^{2+} and Zn^{2+} Ions in Macroalgae with Heteroelement-Doped Carbon-Based Fluorescent Probe

Hui Xu [1,2,3,*], Xin You [3], Yue Lu [3], Peng Liang [3], Zhihui Luo [4], Yiwei Wang [3], Shaoxiao Zeng [1,2,3] and Hongliang Zeng [1,2,3]

[1] Engineering Research Centre of Fujian-Taiwan Special Marine Food Processing and Nutrition, Ministry of Education, Fuzhou 350002, China; zsxfst@fafu.edu.cn (S.Z.); zhlfst@fafu.edu.cn (H.Z.)
[2] Fujian Provincial Key Laboratory of Quality Science and Processing Technology in Special Starch, Fujian Agriculture and Forestry University, Fuzhou 350002, China
[3] College of Food Science, Fujian Agriculture and Forestry University, Fuzhou 350002, China; 3210910056@fafu.edu.cn (X.Y.); luyueeee @fafu.edu.cn (Y.L.); liangpeng0412@fafu.edu.cn (P.L.); wangyw@fafu.edu.cn (Y.W.)
[4] Guangxi Key Laboratory of Agricultural Resources Chemistry and Biotechnology, College of Chemistry and Food Science, Yulin Normal University, Yulin 537000, i, China; zhluo@ylu.edu.cn
* Correspondence: xhuifst@fafu.edu.cn

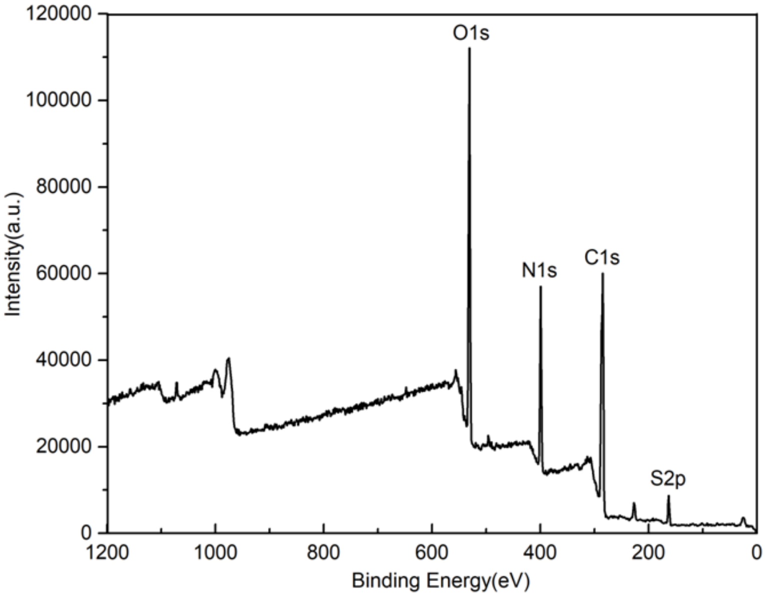

Figure S1. Full XPS spectra of R-CDs.

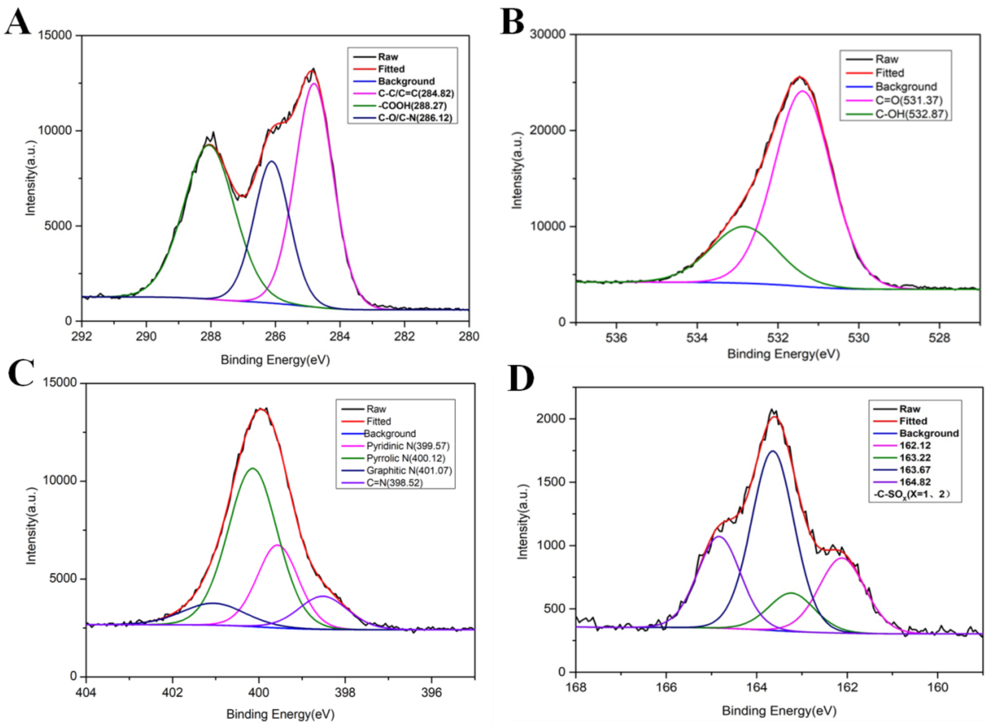

Figure S2. XPS C1s (**A**), O1s (**B**), N1s (**C**) and S2p(**D**) spectra of R-CDs.

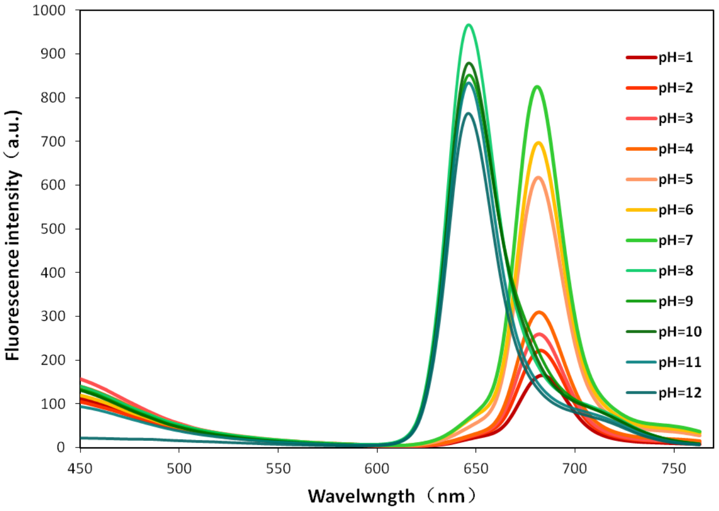

Figure S3. The effect of pH on the fluorescence emission of R-CDs (λ_{Ex} = 420 nm).

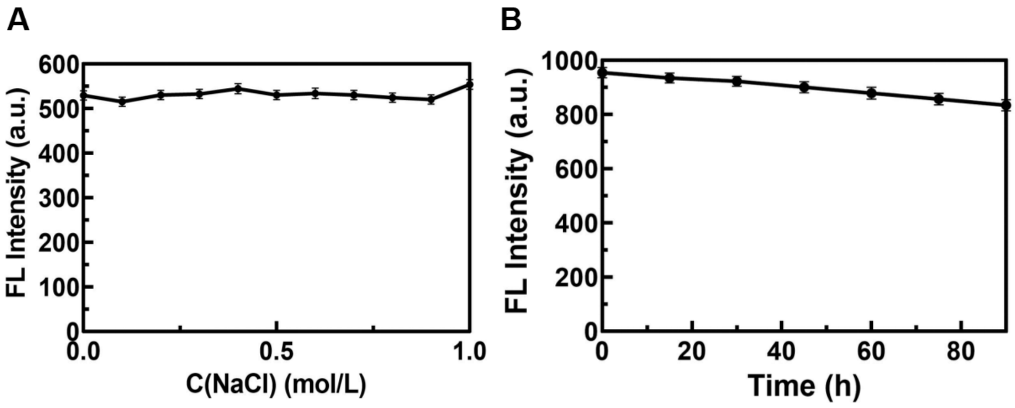

Figure S4. Fluorescence intensities (λ_{Ex} = 420 nm, λ_{Em} = 680 nm) changing of R-CDs in different concentration solutions of NaCl (**A**) and under continuous UV illumination (**B**).

Optimization of R-CDs for Mn^{2+} detection

Due to the pH-dependence of R-CDs, the pH condition was further optimized. The effect of Mn^{2+} on the probe quenching efficiency was investigated in the pH range of 4~10. As shown in Figure S5A, $(F_0\text{-}F)/F_0$ increased with pH of the solution, and the quenching efficiency reached the highest when pH was 7. In alkaline condition, $(F_0\text{-}F)/F_0$ gradually decreased. Therefore, pH=7 was selected as the optimal pH detection condition. The composition and concentration of buffer were further optimized by comparing of different buffers (PBS, HEPES, Tris-HCl, etc.). It was found that HEPES buffer had the highest sensitivity for Mn^{2+} detection, so HEPES was selected as the buffer to keep the pH of the detection environment stable. As can be seen from Figure S5B, R-CDs had the highest quenching efficiency for Mn^{2+} when 0.1 M HEPES (pH=6.8) buffer was used The fluorescence intensity of R-CDs tended to be stable when the response time was 8 min, and there was no significant change after 30 min (Figure S5C). Therefore, the optimal response time was set as 8 min. At this time, the excited electron of R-CDs has filled into the vacant orbital of Mn^{2+} through coordination, causing obvious quenching of fluorescence of R-CDs.

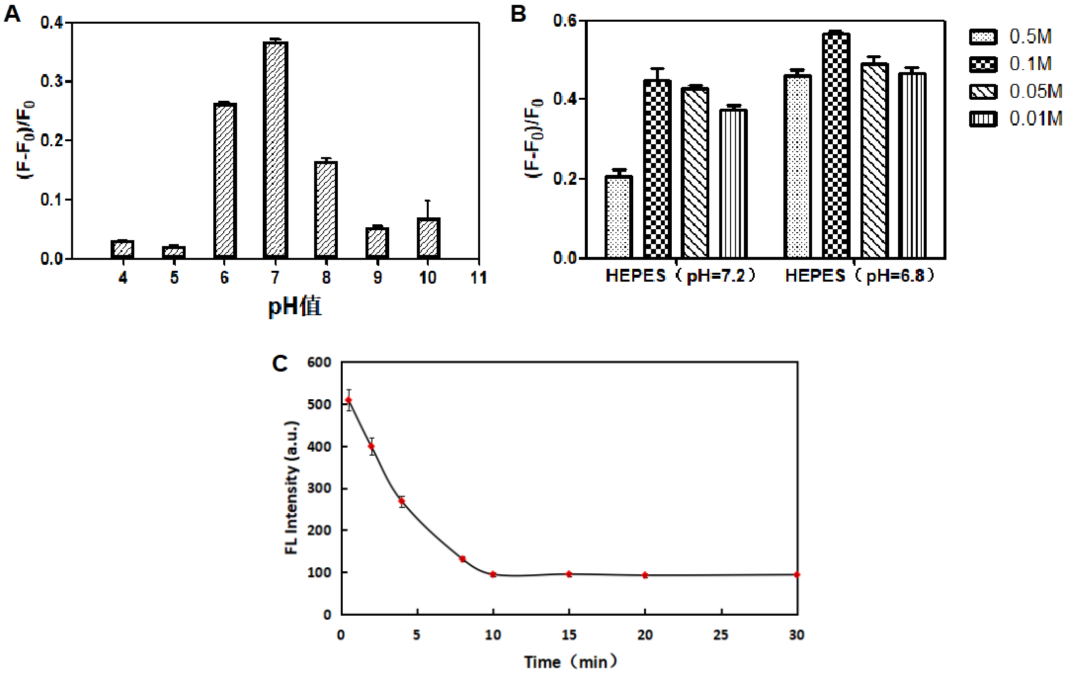

Figure S5. The optimization of pH value (**A**), buffer solution type (**B**) and reaction time (**C**).

Optimization of R-CDs for Zn^{2+} detection

The fluorescence peak at 650 nm was enhanced when pH > 7, while the fluorescence of R-CDs was weakened when pH < 7. Therefore, buffers including HEPES (pH=6.8) and HEPES (pH=7.2) were selected for optimization. As shown in Figure S6A, R-CDs was most sensitive to Zn^{2+} when 0.01 M HEPES buffer (pH=6.8) was used, and the fluorescence signal was significant. As shown in Figure S6B, after Zn^{2+} was added to R-CDs for 7 min, the fluorescence signal reached the maximum value and leveled off. Therefore, 7 min was taken as the optimal response time, when the intramolecular charge transfer was sufficient, which was conducive to the formation of the fluorescence peak at 650 nm.

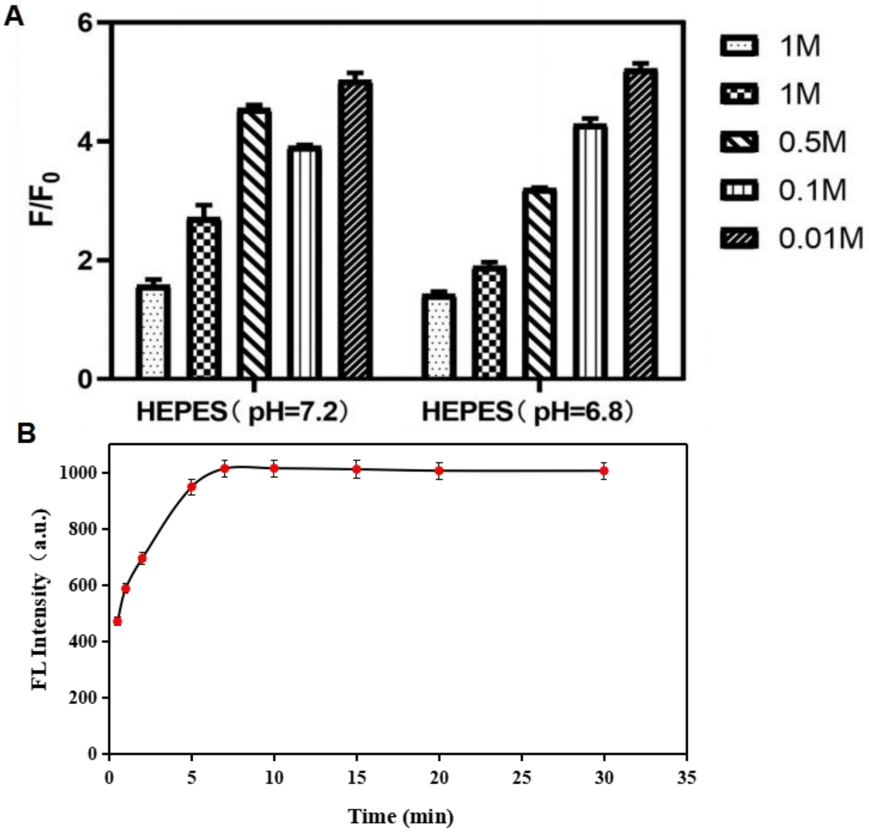

Figure S6. The optimization of buffer solution type (**A**) and reaction time (**B**).
